# Supplementary material for: Human Fis1 regulates mitochondrial dynamics through inhibition of the fusion machinery
Source: EMBO J. 2019 Mar 6;38(8):e99748. doi: 10.15252/embj.201899748 (PMC6463211; doi:10.15252/embj.201899748)
Supplement: Supplementary file 1 — Appendix [file EMBJ-38-e99748-s001.pdf]

Appendix Figures

Human Fis1 regulates mitochondrial dynamics through inhibition of the fusion machinery

Rong Yu[1], Shao-Bo Jin[2], Urban Lendahl[2], Monica Nistér[1,3]*, and Jian Zhao[1,3]*

[1] Department of Oncology-Pathology, Karolinska Institutet, CCK R8:05, Karolinska University Hospital Solna, SE-171 76 Stockholm, Sweden;

[2] Department of Cell and Molecular Biology, Karolinska Institutet, SE-171 77 Stockholm, Sweden

[3] These authors share senior responsibility

Table of Contents

Item	Title	Page No.
Appendix Figure S1	Generation of *DRP1* knockout ($Drp1^{-/-}$) 293T cell lines by CRISPR/Cas9 gene editing (related to Fig 1).	2
Appendix Figure S2	Generation of *DRP1* knockout ($Drp1^{-/-}$) HeLa cell lines by CRISPR/Cas9 gene editing (related to Fig EV1).	3
Appendix Figure S3	Generation of human *FIS1* knockout ($hFis1^{-/-}$) 293T cell lines by CRISPR/Cas9 gene editing (related to Fig 4).	4
Appendix Figure S4	Generation of *DRP1/OPA1* double knockout ($Drp1/OPA1^{DKO}$) 293T cell lines by CRISPR/Cas9 gene editing (related to Fig 8).	5

Appendix Figures

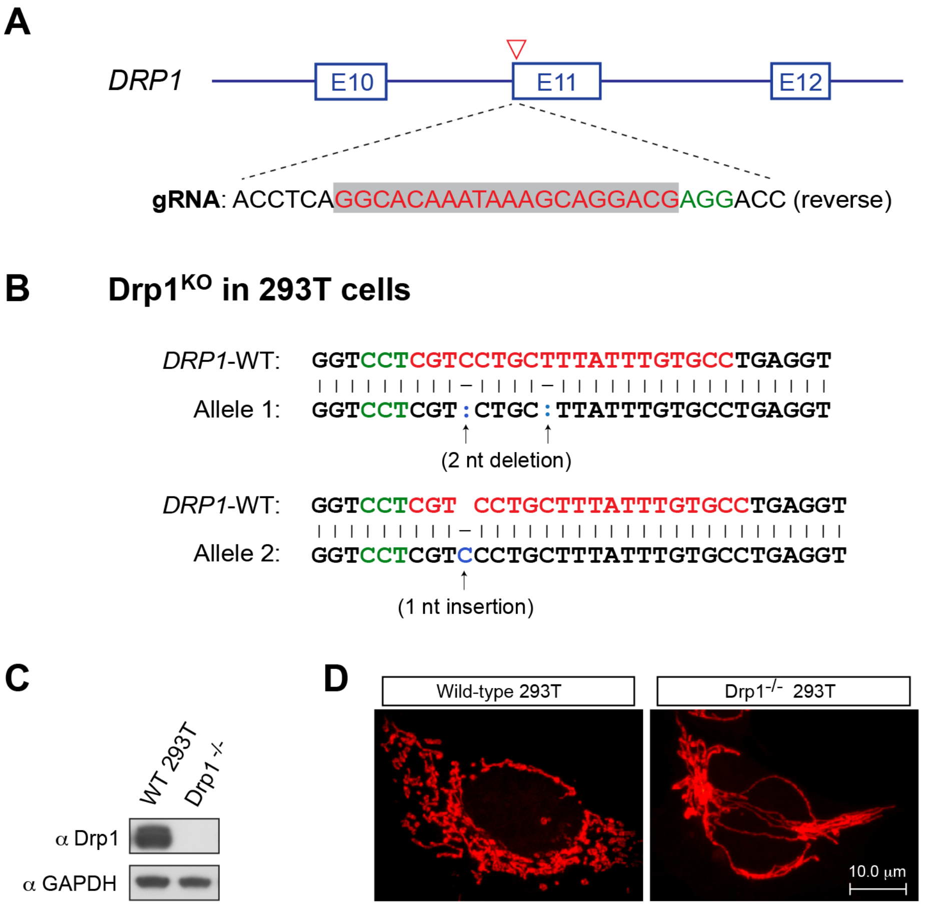

Appendix Figure S1. Generation of *DRP1* knockout (*Drp1* $^{-/-}$) 293T cell lines by CRISPR/Cas9 gene editing (related to Fig 1).

A. The sequence and location of single guide RNA (gRNA, red) targeted to the human DRP1 gene and the corresponding protospacer adjacent motif (PAM, green) required for CRISPR/Cas9 gene targeting are shown.

B. Sequence alignments around the edited gRNA targeting sites of wild-type (WT) and mismatches (blue) in the *DRP1* knockout clonal cell line. The data were obtained from PCR cloning followed by sequencing.

C. The single cell colonies derived and expanded from *Drp1*$^{-/-}$ 293T cells were confirmed by Western blot analysis as indicated.

D. Genetic ablation of Drp1 resulted in a super-fused mitochondrial phenotype as confirmed by confocal microscopy (stained with MitoTracker Red).

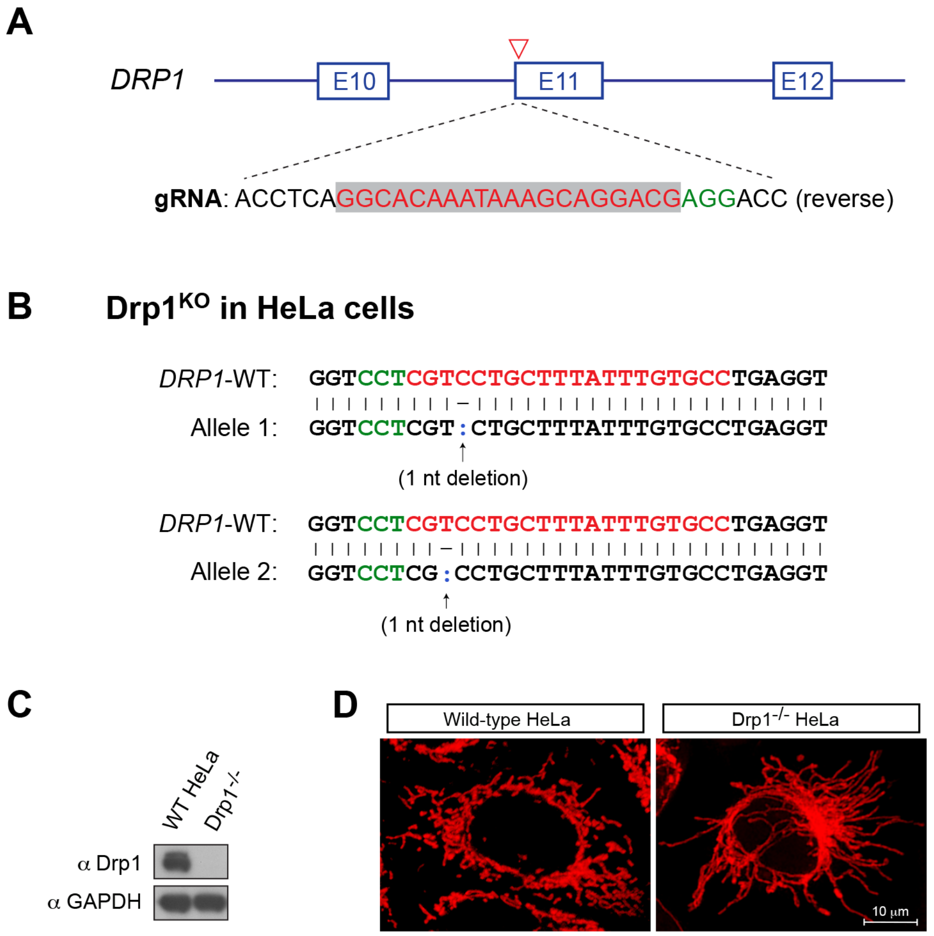

Appendix Figure S2. Generation of *DRP1* knockout (*Drp1* $^{-/-}$) HeLa cell lines by CRISPR/Cas9 gene editing (related to Fig EV1).

A. The sequence and location of single guide RNA (gRNA, red) targeted to the human DRP1 gene and the corresponding protospacer adjacent motif (PAM, green) required for CRISPR/Cas9 gene targeting are shown.

B. Sequence alignments around the edited gRNA targeting sites of wild-type (WT) and mismatches (blue) in the *DRP1* knockout clonal cell line. The data were obtained from PCR cloning followed by sequencing.

C. The single cell colonies derived and expanded from *Drp1*$^{-/-}$ HeLa cells were confirmed by Western blot analysis as indicated.

D. Genetic ablation of Drp1 resulted in a super-fused mitochondrial phenotype as confirmed by confocal microscopy (stained with MitoTracker Red).

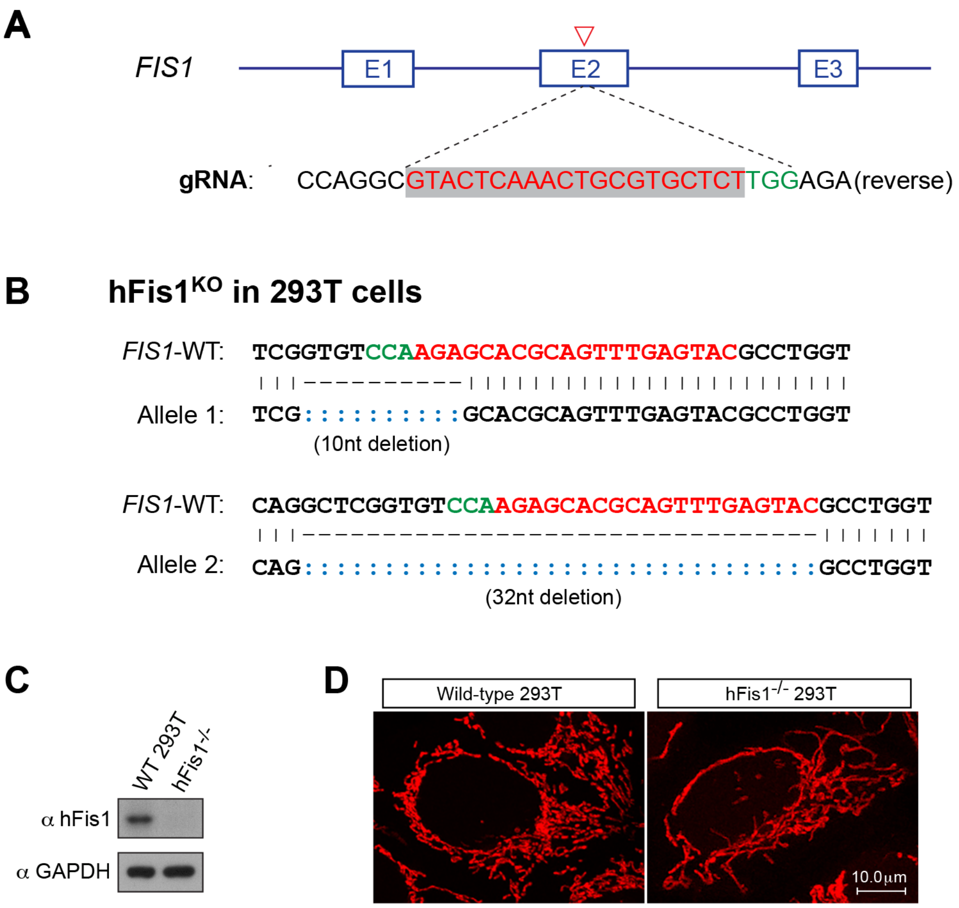

Appendix Figure S3. Generation of human *FIS1* knockout (*hFis1*$^{-/-}$) 293T cell lines by CRISPR/Cas9 gene editing (related to Fig 4).

A. The sequence and location of single guide RNA (gRNA, red) targeted to the human Fis1 gene and the corresponding protospacer adjacent motif (PAM, green) required for CRISPR/Cas9 gene targeting are shown.

B. Sequence alignments around the edited gRNA targeting sites of wild-type (WT) and mismatches (blue) in the *hFIS1* knockout clonal cell line. The data were obtained from PCR cloning followed by sequencing.

C. The single cell colonies derived and expanded from *hFis1*$^{-/-}$ 293T cells were confirmed by Western blot analysis as indicated.

D. Genetic ablation of hFis1 resulted in a tubular mitochondrial phenotype as confirmed by confocal microscopy (stained with MitoTracker Red).

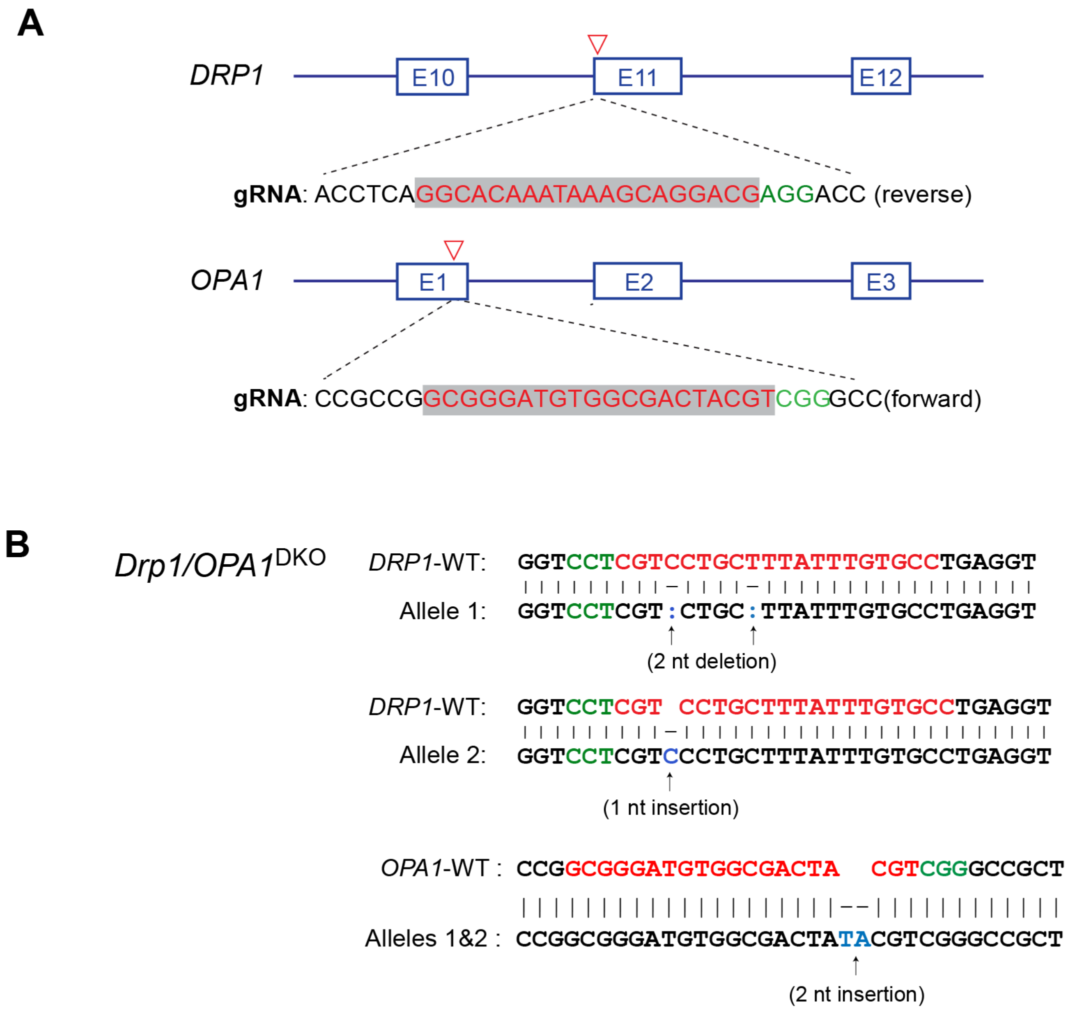

Appendix Figure S4. Generation of *DRP1/OPA1* double knockout (*Drp1/OPA1*^{DKO}) 293T cell lines by CRISPR/Cas9 gene editing (related to Fig 8).

A. The sequences and locations of single guide RNAs (gRNA, red color) targeted to the human DRP1 and OPA1 genes and the corresponding protospacer adjacent motif (PAM, green color) required for CRISPR/Cas9 gene targeting are shown. The *DRP1* KO is as described in Appendix Figure S1.

B. Sequence alignments around the edited gRNA targeting sites of wild-type (WT) and mismatches (blue color) in the *DRP1/OPA1* double knockout clonal cell line. The data were obtained from PCR cloning followed by sequencing.
