# Supplementary material for: Human Fis1 regulates mitochondrial dynamics through inhibition of the fusion machinery
Source: EMBO J. 2019 Mar 6;38(8):e99748. doi: 10.15252/embj.201899748 (PMC6463211; doi:10.15252/embj.201899748)
Supplement: Supplementary file 2 — Expanded View Figures PDF [file EMBJ-38-e99748-s002.pdf]

Expanded View Figures

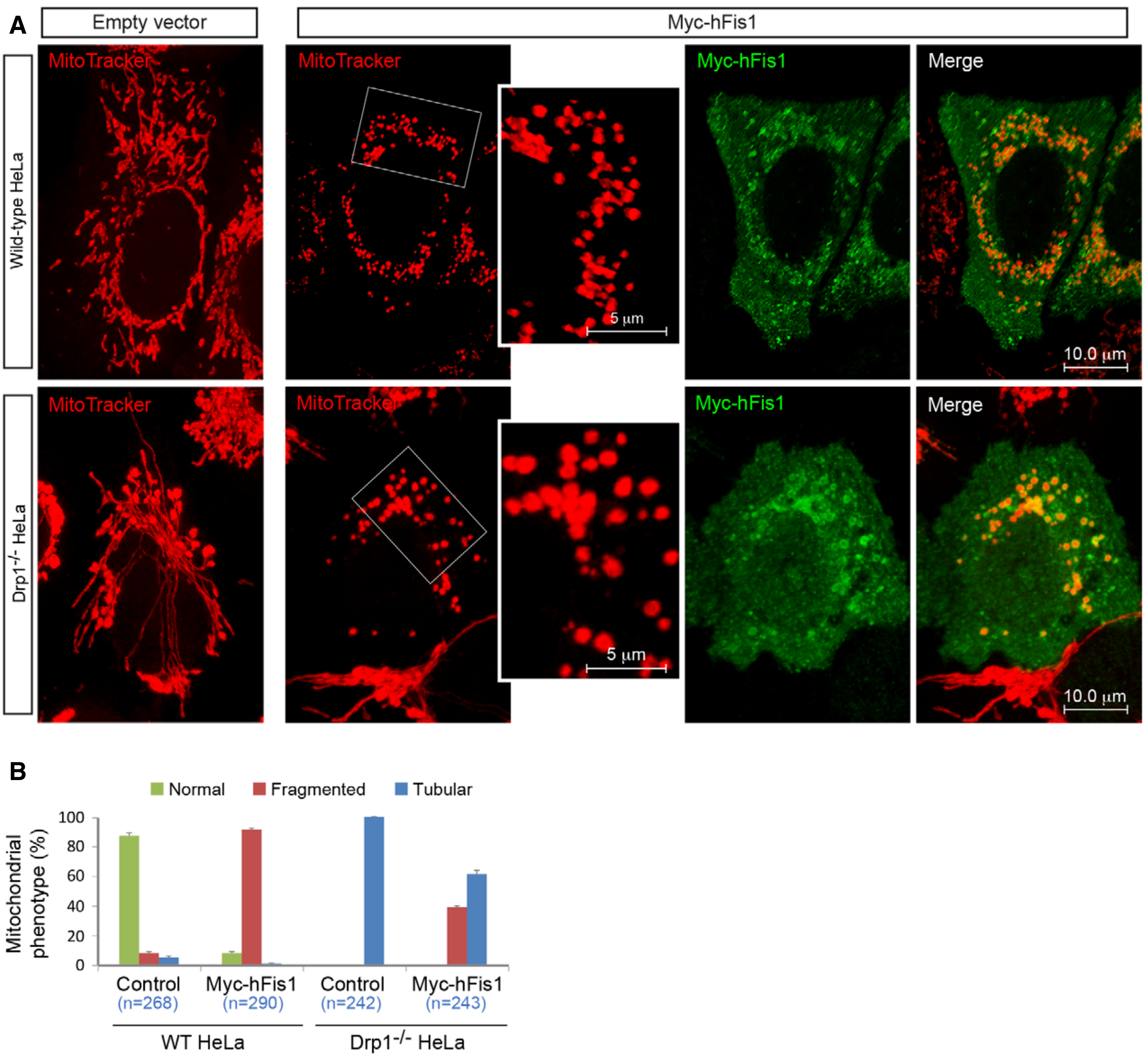

Figure EV1. Drp1 is largely dispensable for mitochondrial fragmentation induced by hFis1 in HeLa cells (related to Fig 1).

A Confocal images of mitochondrial morphology in wild-type and Drp1$^{-/-}$ HeLa cells transfected with empty vector (left panel) and Myc-hFis1 (right panel), stained with MitoTracker (red) followed by immunostaining with anti-Myc antibody (green). Insets represent high magnification views of the boxed areas.

B Percentages (mean ± SEM) of cells with indicated mitochondrial morphologies in wild-type and Drp1$^{-/-}$ HeLa cells transfected with empty vector (control) or Myc-hFis1 in three independent experiments (*n* represents the number of cells analyzed).

 © 2019 The Authors

 © 2019 The Authors

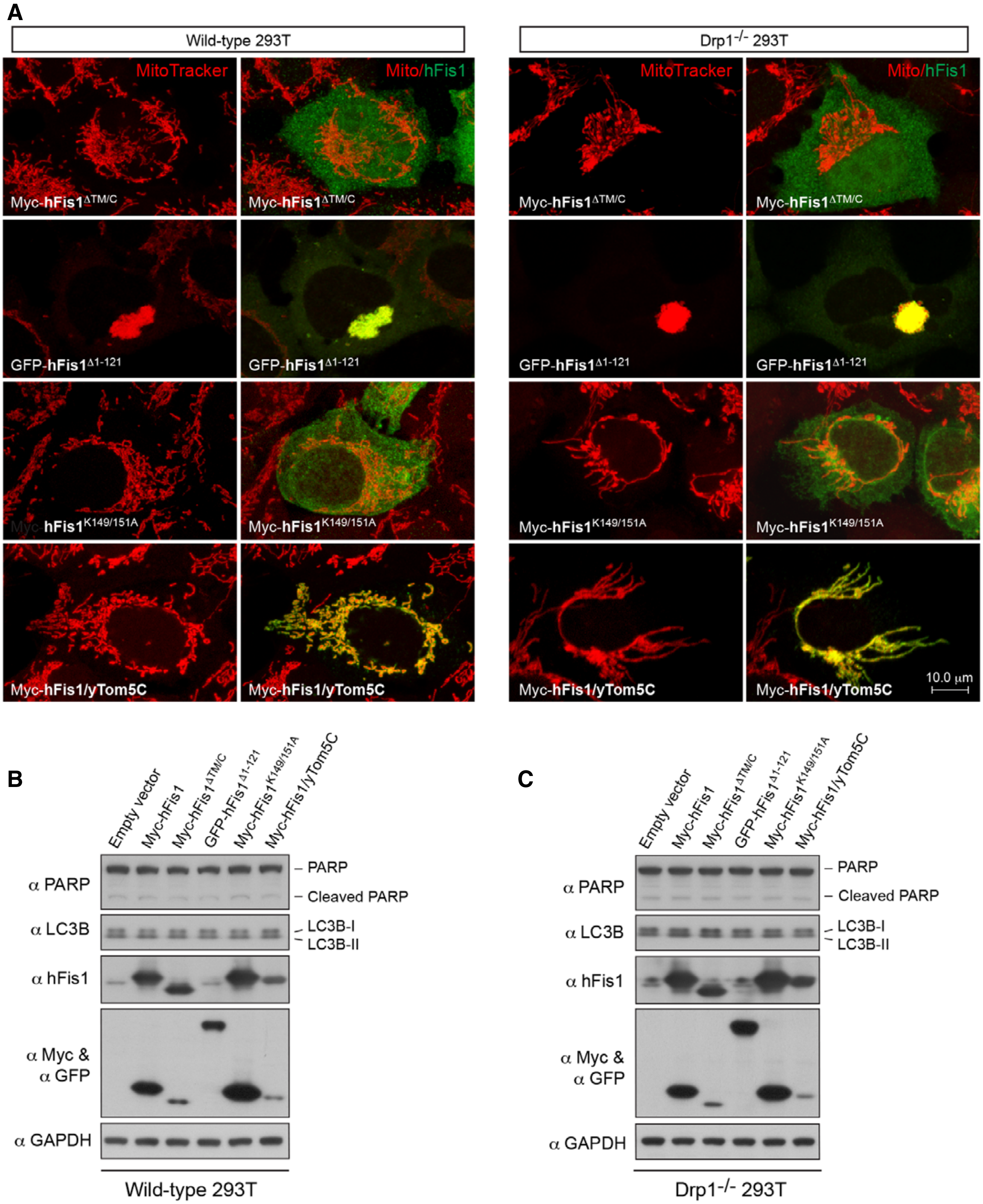

Figure EV2. Subcellular localization and mitochondrial phenotypes of hFis1 mutants; hFis1 overexpression does not induce apoptosis and autophagy in WT and Drp1$^{-/-}$ 293T cells (related to Fig 1).

A Representative confocal images of mitochondrial morphology in wild-type (left panel) and Drp1$^{-/-}$ 293T cells (right panel) transfected with different hFis1 mutants as indicated, stained with MitoTracker (red) followed by immunostaining with anti-Myc (green) or anti-GFP antibody (green).

B, C Overexpression of WT hFis1 and mutants does not affect apoptosis and autophagy as analyzed by immunoblotting with PARP and LC3B antibodies. WT 293T (B) and Drp1$^{-/-}$ 293T (C) cells were transiently transfected with 0.5 µg of empty vector, Myc-hFis1, Myc-hFis1$^{\Delta TM/C}$, GFP-hFis1$^{\Delta 1-121}$, Myc-hFis1$^{K149/151A}$, or Myc-hFis1/yTom5C plasmid. Cells were harvested after transfection for 20 h and analyzed by Western blotting with the indicated antibodies.

Source data are available online for this figure.

© 2019 The Authors

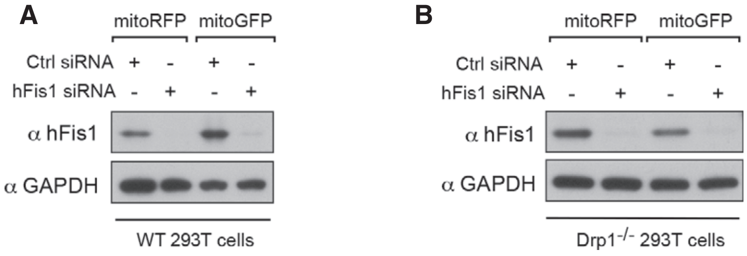

Figure EV3. hFis1 impairs mitochondrial fusion in WT and Drp1$^{-/-}$ 293T cells—control experiments (related to Figs 5 and 6).

A Knockdown of hFis1 by siRNA in wild-type 293T cells with stable expression of either mitoGFP or mitoRFP is confirmed by Western blotting analysis.

B Knockdown of hFis1 by siRNA in Drp1$^{-/-}$ 293T cells with stable expression of either mitoGFP or mitoRFP is confirmed by Western blotting analysis.

Source data are available online for this figure.

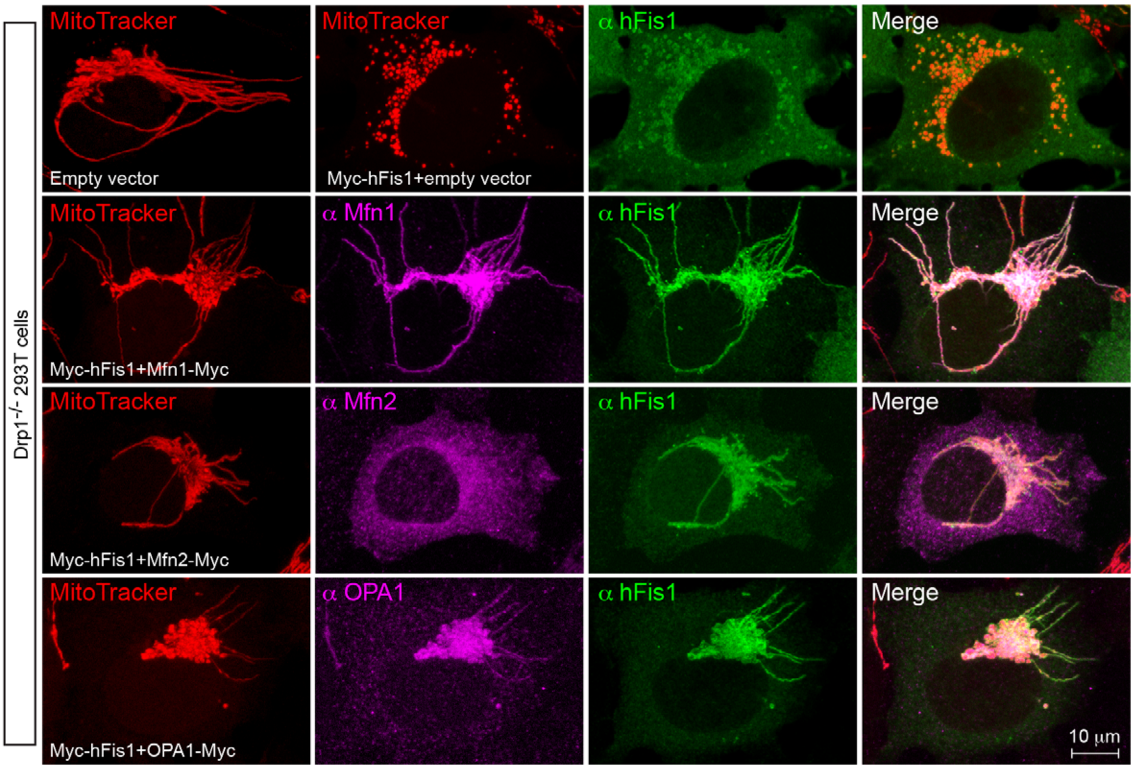

Figure EV4. Effect of the pro-fusion GTPases (Mfns and OPA1) on hFis1-induced mitochondrial fragmentation in Drp1$^{-/-}$ cells (related to Fig 7G).

Representative confocal images of the mitochondrial phenotype in Drp1$^{-/-}$ 293T cells co-transfected with Myc-hFis1 and either empty vector, Mfn1-Myc, Mfn2-Myc, or OPA1-Myc as indicated, and stained with MitoTracker (red) followed by immunostaining with anti-hFis1 (green) and either anti-Mfn1, Mfn2, or OPA1 antibodies (pink). The data indicate an inhibitory/competitive effect of pro-fusion GTPase proteins on the hFis1-mediated mitochondrial fragmentation in Drp1-deficient cells.

 © 2019 The Authors

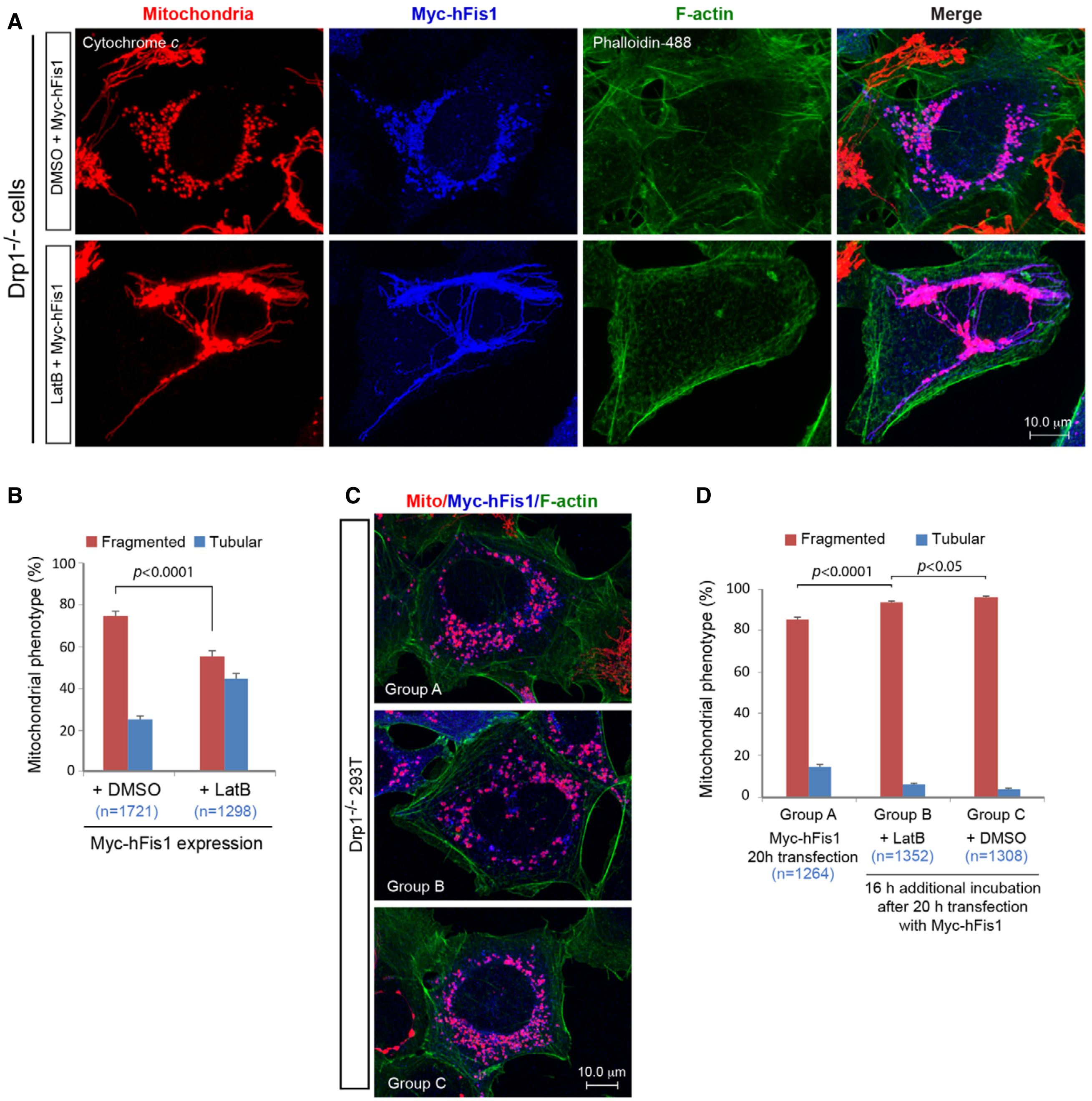

Figure EV5. Filamentous actin (F-actin) is potentially involved in the for mitochondrial fragmentation induced by hFis1 overexpression in Drp1-deficient cells (related to the last two paragraphs of the Results section).

A Confocal images of mitochondrial morphology in Drp1$^{-/-}$ 293T cells transfected with Myc-hFis1 or treated with DMSO (control) for 4 h and then treated with DMSO (control) or 0.25 μM latrunculin B (LatB) for an additional 16 h, followed by immunostaining with anti-Myc antibody for Myc-hFis1 (blue), anti-cytochrome *c* antibody for mitochondria (red), and Alexa Fluor® 488 Phalloidin for F-actin (green).

B Percentages (mean ± SEM) of cells with indicated mitochondrial morphologies in (A). Data were collected from three independent experiments (*n* represents the number of cells analyzed).

C Confocal images of mitochondrial morphology in Drp1$^{-/-}$ 293T cells with Myc-hFis1 overexpression and treated under different conditions as indicated. Three sets of Drp1$^{-/-}$ 293T cells were transfected with Myc-hFis1 for 20 h. Group A was harvested directly at 20 h posttransfection, whereas Groups B and C were subsequently treated with 0.25 μM LatB or DMSO (control) for 16 h, then harvested and immunostained with anti-Myc antibody, and analyzed under the confocal microscope.

D Percentages (mean ± SEM) of cells with indicated mitochondrial morphologies in (C). Data were collected from three independent experiments (*n* represents the number of cells analyzed).

© 2019 The Authors
