# Supplementary figures and images for: Human Fis1 regulates mitochondrial dynamics through inhibition of the fusion machinery
Source: EMBO J. 2019 Mar 6;38(8):e99748. doi: 10.15252/embj.201899748 (PMC6463211; doi:10.15252/embj.201899748)

Fig. EV2B

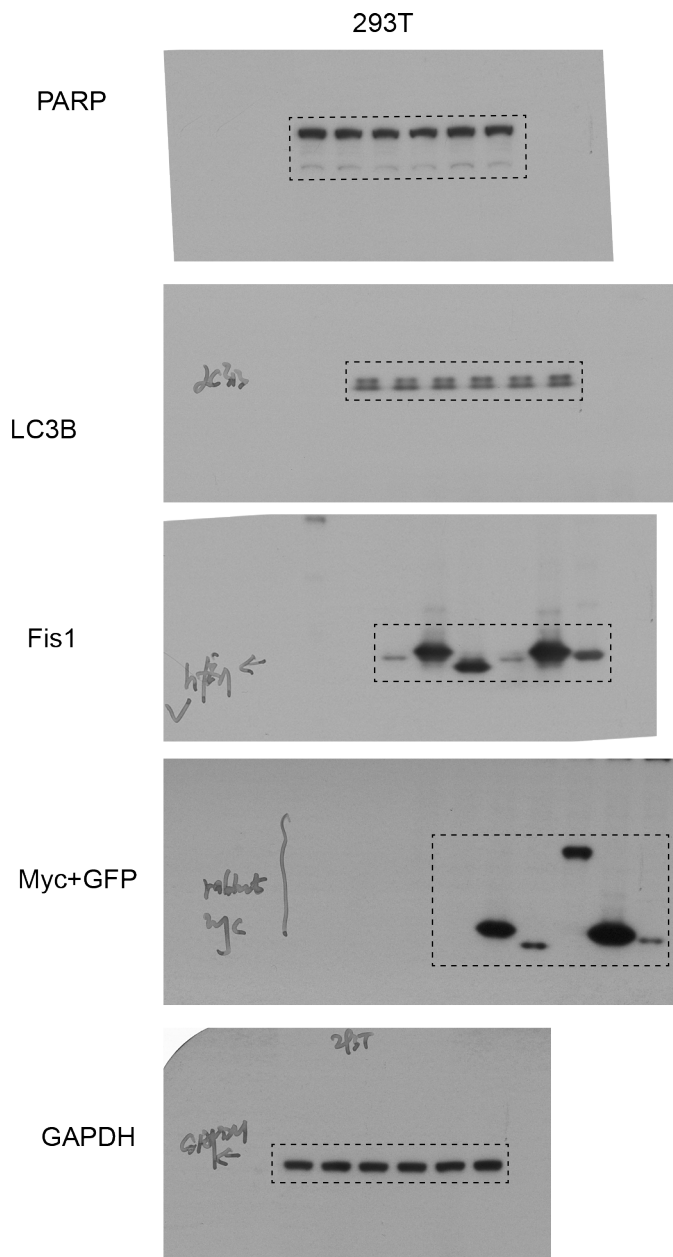

Fig. EV2C

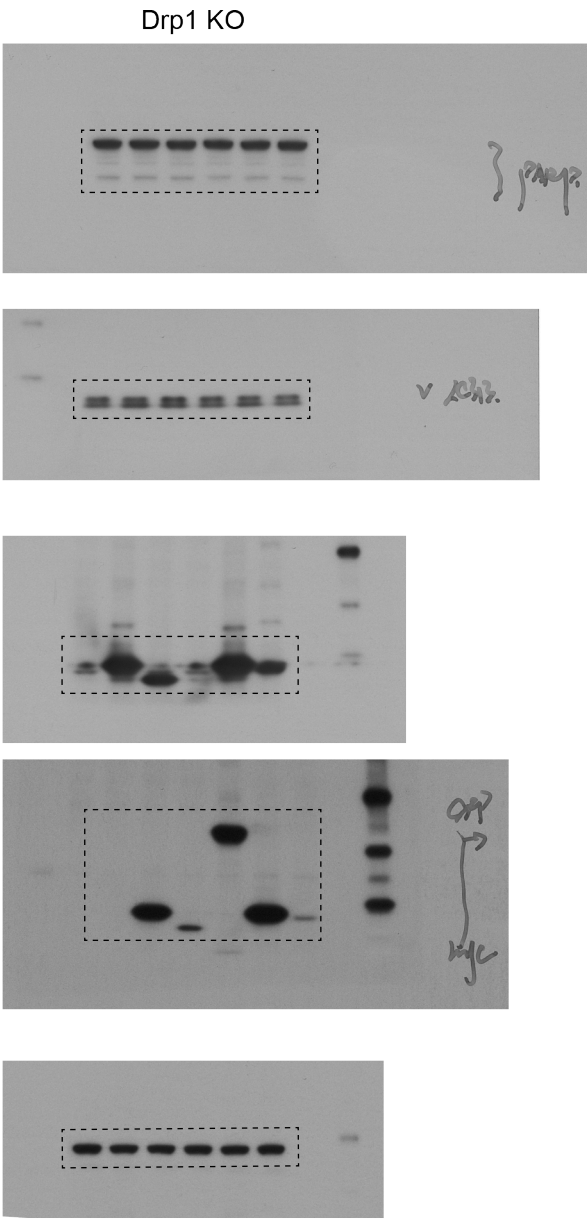

Supplement: Supplementary file 3 — Source Data for Expanded View [file EMBJ-38-e99748-s008.zip › Figure_EV2_Source_Data.pdf]

Fig. EV3A

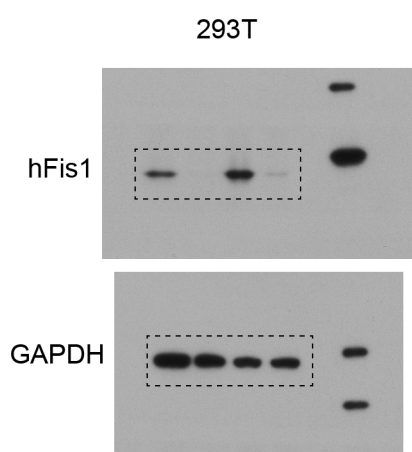

Fig. EV3B

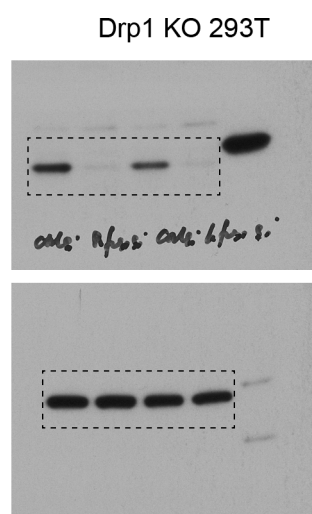

Supplement: Supplementary file 3 — Source Data for Expanded View [file EMBJ-38-e99748-s008.zip › Figure_EV3_Source_Data.pdf]

Fig. 2B

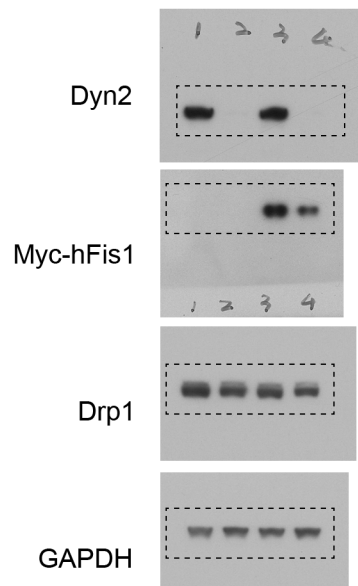

Fig. 2E

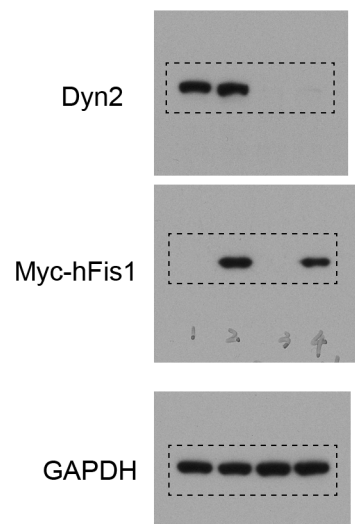

Supplement: Supplementary file 5 — Source Data for Figure 2 [file EMBJ-38-e99748-s003.pdf]

Fig.3D

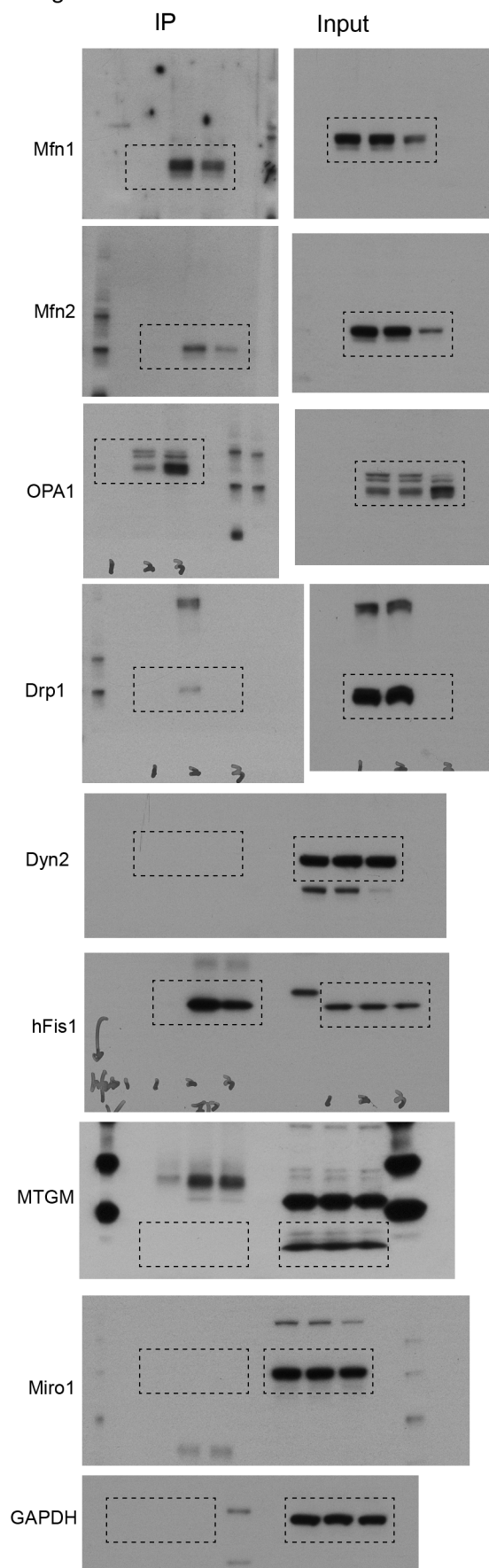

Fig.3E and F

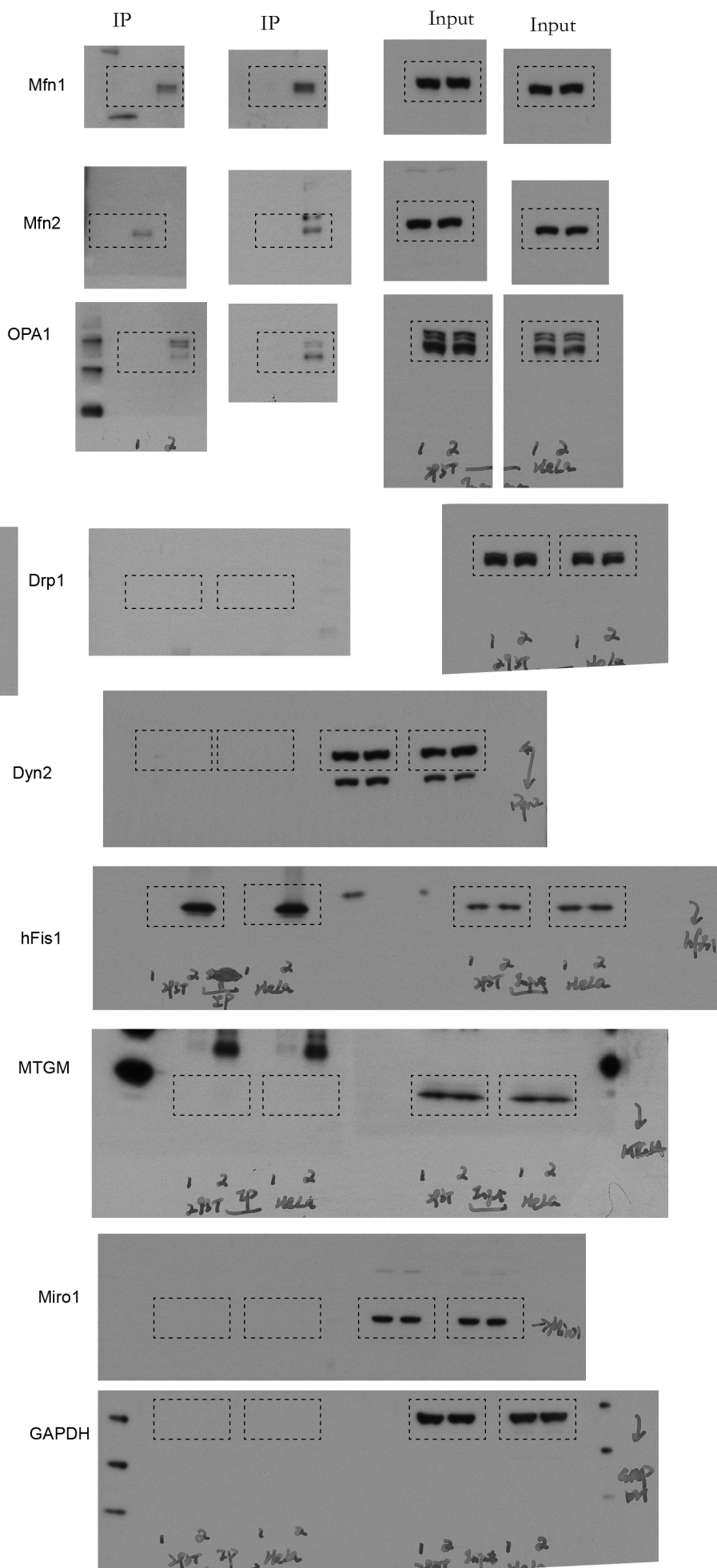

Fig.3G

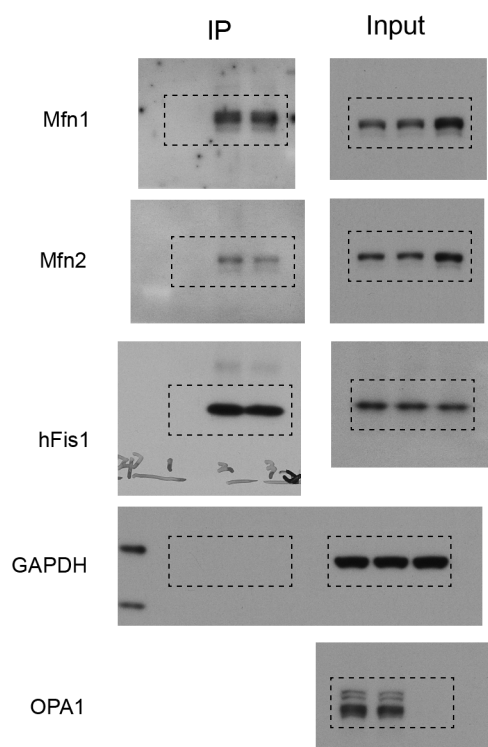

Fig.3H

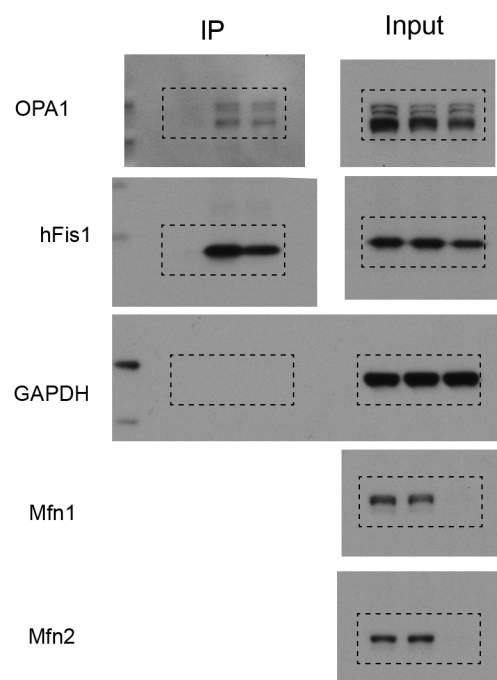

Fig.3I

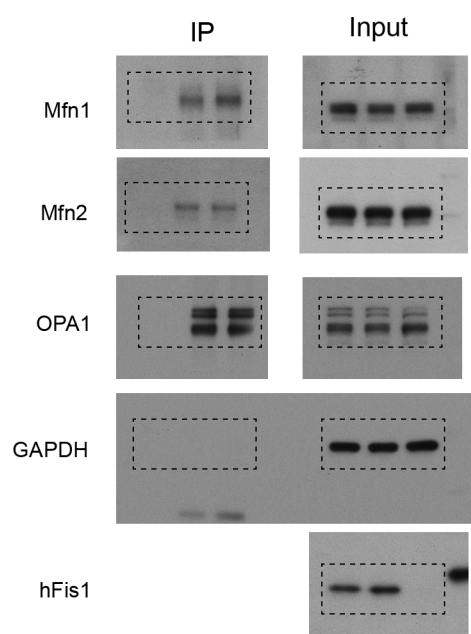

Fig.3J

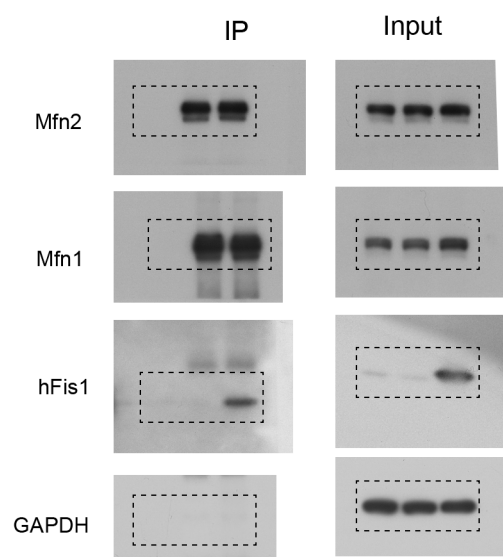

Supplement: Supplementary file 6 — Source Data for Figure 3 [file EMBJ-38-e99748-s004.pdf]

Fig. 4B

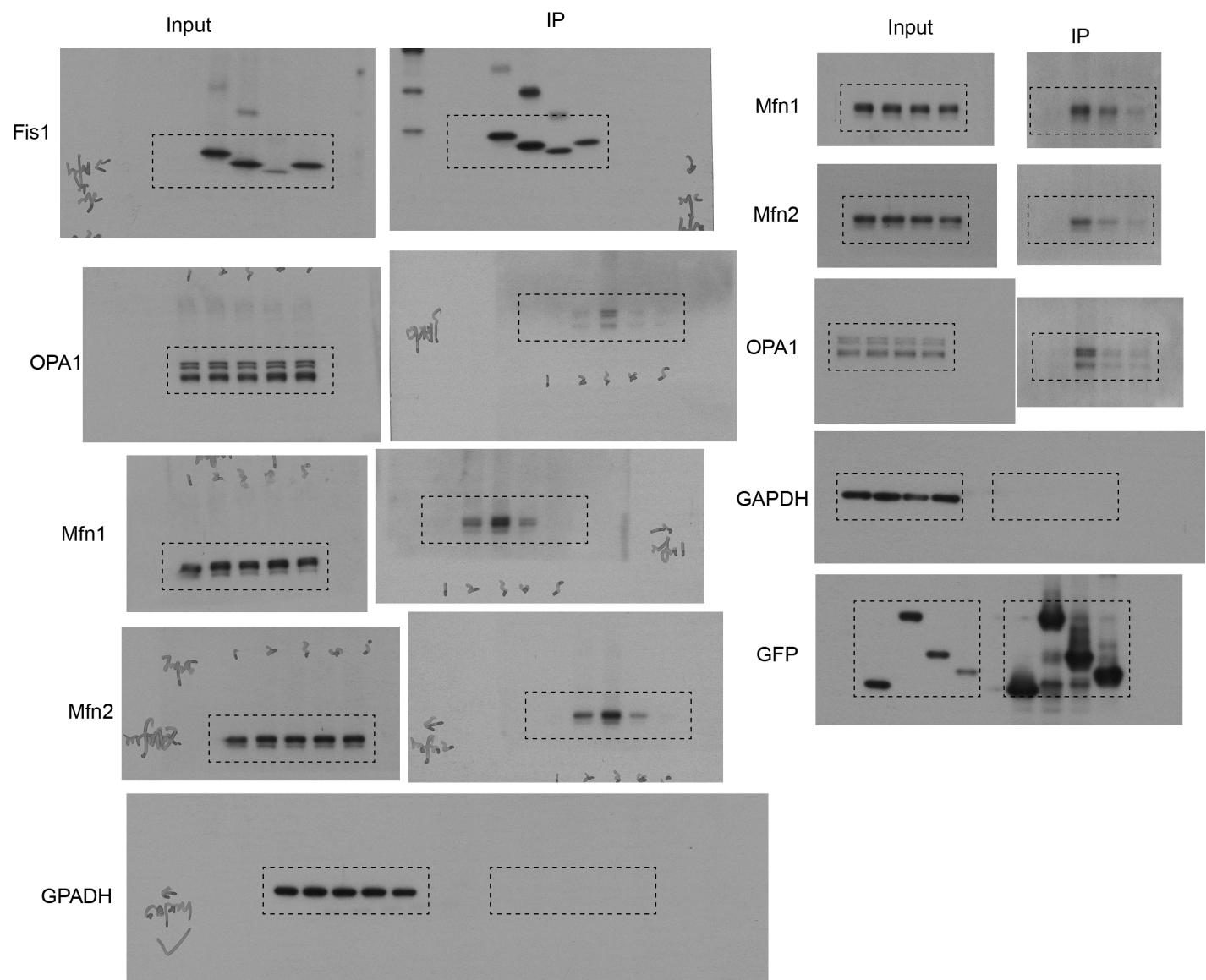

Supplement: Supplementary file 7 — Source Data for Figure 4 [file EMBJ-38-e99748-s005.pdf]

Fig. 7

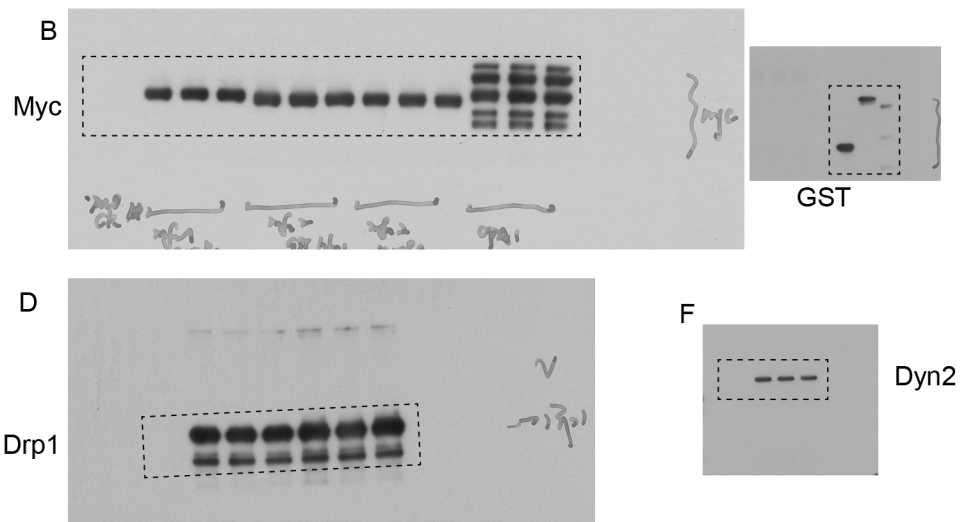

Supplement: Supplementary file 8 — Source Data for Figure 7 [file EMBJ-38-e99748-s006.pdf]

Fig. 8A

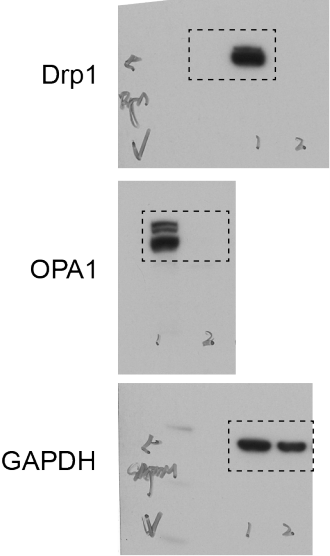

Fig. 8E

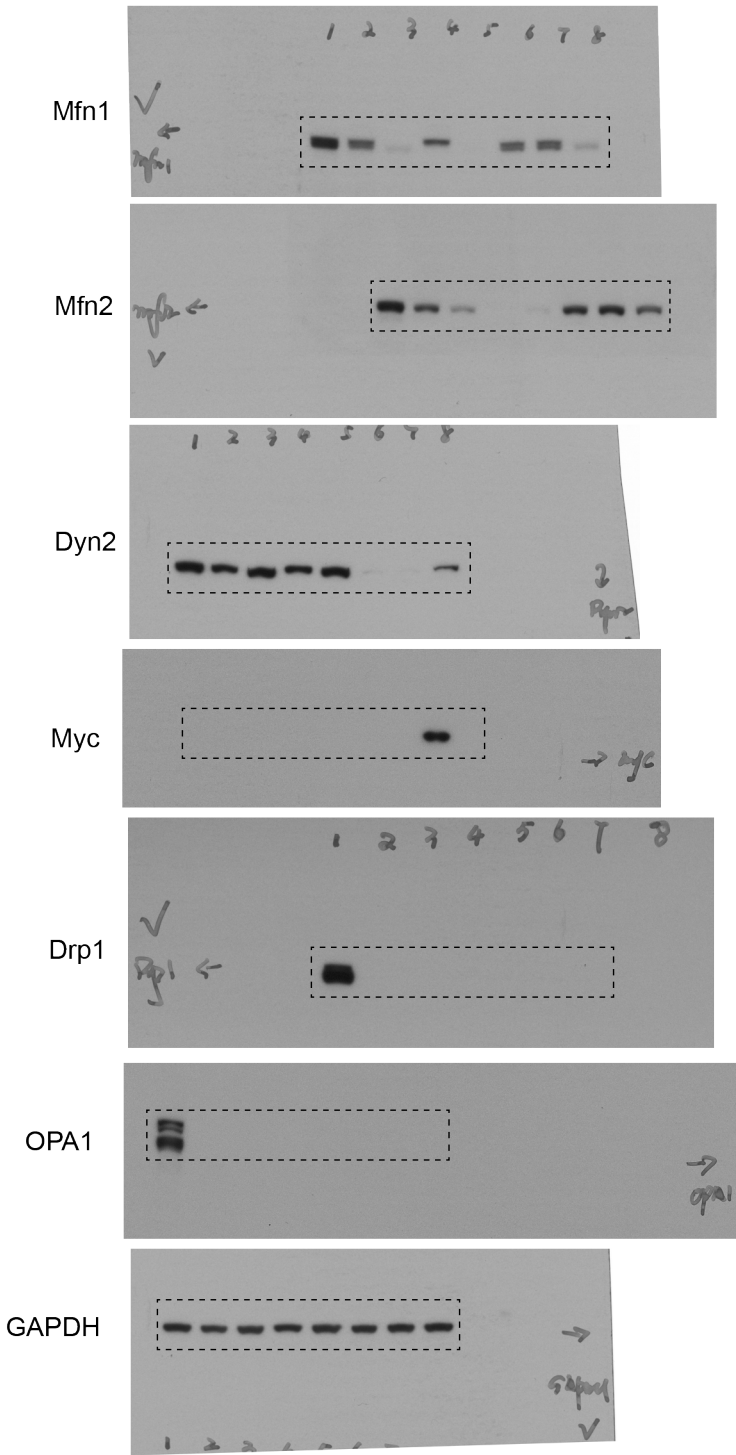

Supplement: Supplementary file 9 — Source Data for Figure 8 [file EMBJ-38-e99748-s007.pdf]
